# Long-Term Nitrogen Fertilization Increases Soil Organic Carbon and Wheat Yields on Purple Soil in China

**DOI:** 10.3390/plants14182866

**Published:** 2025-09-15

**Authors:** Jasim Iqbal, Zhiyuan Yao, Syed Turab Raza, Hassan Iqbal, Bo Zhu

**Affiliations:** 1 Key Laboratory of Mountain Surface Processes and Ecological Regulation, Institute of Mountain Hazards and Environment, Chinese Academy of Sciences, Chengdu 610041, China; jasimkhan38@imde.ac.cn (J.I.); bzhu@imde.ac.cn (B.Z.); 2 University of Chinese Academy of Sciences, Beijing 100049, China; 3 Yunnan Key Laboratory of Meteorological Disasters and Climate Resources in the Greater Mekong Sub Region, School of Earth Sciences, Yunnan University, Kunming 650500, China; 4 Yunnan Key Laboratory of Plant Reproductive Adaptation and Evolutionary Ecology, Institute of Biodiversity, School of Ecology and Environmental Science, Yunnan University, Kunming 650500, China; 5 State Key Laboratory of Desert and Oasis Ecology, Xinjiang Institute of Ecology and Geography, Chinese Academy of Sciences, Urumqi 830011, China; hassan@ms.xjb.ac.cn

**Keywords:** soil organic carbon, wheat production, soil fertility, nutrient cycling, environmental pollution

## Abstract

Synthetic nitrogen (N) fertilization is essential for global food security, but often over-applied, causing environmental pollution. Identifying the optimal N application rate that maximizes crop productivity while enhancing key soil properties remains essential for sustainable agriculture. Three treatments from a 21-year field experiment conducted on purple soil in the Sichuan Basin, China, were studied: unfertilized control (CK), moderate synthetic N application (NPK; 130 kg ha^−1^), and higher synthetic N application (HNPK; 170 kg ha^−1^). The results showed that NPK and HNPK increased key soil properties compared to CK, with total N increasing by 44%, microbial biomass N by 48%, microbial biomass C by 81%, and soil organic C by 33% (*p* < 0.05). Both NPK and HNPK significantly enhanced plant N and C accumulation compared to CK (*p* < 0.05), resulting in substantial increases in grain yield (436%) and biomass yield (319%). Notably, NPK and HNPK achieved comparable enhancements in soil properties, N use efficiency, and crop productivity (*p* < 0.05). Additionally, Random Forest model (R^2^ = 0.91) identified soil N pools and plant N uptake as primary yield predictors. These findings suggest that moderate N application achieves comparable crop productivity and soil enhancement benefits to higher application rates, supporting resource-efficient agricultural practices that contribute to sustainable intensification in subtropical agroecosystems.

## 1. Introduction

Global food security faces unprecedented challenges as the world population approaches 8 billion people, with agricultural systems under increasing pressure to meet growing food demands while maintaining environmental sustainability [1,2]. Modern intensive agriculture has become heavily dependent on synthetic fertilizer inputs to achieve the high crop yields necessary for feeding this expanding population [3]. Among essential plant nutrients, nitrogen (N) represents the most critical macronutrient limiting crop production in agricultural systems worldwide, with synthetic N fertilizers now supporting approximately 48% of global food production, particularly in intensive farming systems across Asia, Europe, and North America [4,5]. However, the widespread adoption of high-input farming practices has led to excessive N fertilizer application in many regions, resulting in severe environmental consequences, including groundwater contamination, eutrophication of aquatic ecosystems, emission of nitrous oxide (N_2_O) contributing to climate change, and loss of soil biodiversity [6,7,8,9]. Additionally, the inefficient use of N fertilizers poses significant risks to ecosystem integrity, as excess N can disrupt natural biogeochemical cycles, promote algal blooms in water bodies, and contribute to the formation of oceanic dead zones where aquatic life cannot survive [10,11]. Therefore, optimizing N fertilization is essential to mitigate environmental impacts while sustaining crop productivity and enhancing soil properties.

Past research examining N fertilization effects on key soil properties has exhibited contradictory findings that complicate the development of evidence-based management recommendations. For instance, several studies demonstrated the beneficial effects of appropriate N fertilization, showing enhanced soil organic carbon (SOC) accumulation, improved microbial biomass and activity, and increased nutrient cycling efficiency in agricultural systems [12,13]. SOC enhancement represents a critical component of climate change mitigation strategies, as increased soil C sequestration reduces atmospheric carbon dioxide (CO_2_) concentrations while simultaneously improving soil fertility and structure [14,15,16]. Such positive outcomes were particularly evident in long-term cropping systems, where moderate N fertilization significantly increased SOC content and microbial biomass in wheat–maize rotations, with improvements attributed to enhanced crop residue production and root biomass input [17,18]. Furthermore, N fertilization enhanced soil aggregation and water-holding capacity through increased organic matter content and enhanced microbial activity in Chinese agricultural soils [19].

Conversely, meta-analyses conducted in wheat–maize systems have documented negative impacts of N fertilization on soil properties, including acidification, reduction in soil pH, and disruption of soil nutrient balance [20,21]. Such detrimental effects were particularly pronounced under excessive application rates, where studies have demonstrated significant soil acidification and reduced beneficial microbial populations in intensive cropping systems [22,23]. Additionally, excessive N fertilization compounds soil degradation by simultaneously increasing the risk of nutrient leaching and gaseous N losses, creating both on-site soil deterioration and off-site environmental contamination [24,25]. The divergent findings in the literature restrict our understanding of identifying optimal N fertilization rates that can achieve productivity and soil maintenance goals without excessive application that could lead to environmental pollution and exacerbate climate change. Addressing this knowledge gap is particularly critical in intensive subtropical agricultural regions where determining the precise balance between sufficient nutrient supply and environmental protection remains challenging.

The Yangtze River basin in China covers 1.8 million km^2^, making it the world’s third-largest river system by drainage area [26]. This region supports a population exceeding 400 million inhabitants and serves as a critical agricultural zone, contributing approximately 50–75% of China’s total agricultural production [16]. The intensive agricultural practices in the upper reaches of the Yangtze River basin, characterized by extensive application of synthetic fertilizers to support crop cultivation, have resulted in significant agricultural non-point source pollution, particularly elevated N contamination throughout the watershed [27]. Therefore, adopting knowledge-driven sustainable management strategies is crucial for maintaining soil, ecosystem services, and agricultural productivity in the Yangtze River region. In this framework, this study evaluates the effects of different synthetic N fertilization rates on key soil properties and wheat productivity in a long-term wheat–maize rotation system under a subtropical monsoon climate.

We aim to identify the optimal N fertilization rate that can achieve soil maintenance goals while sustaining high wheat productivity. We hypothesized that both moderate (130 kg ha^−1^) and higher (170 kg ha^−1^) rates of synthetic N fertilizer would achieve comparable soil properties and yield enhancement benefits. Specifically, our objectives are to (1) quantify soil N and C pools under moderate and higher N fertilization regimes; (2) evaluate their impacts on N and C accumulation based on different components of plants along with yields; and (3) assess the relationships between soil N and C pools, total N and C accumulation by plants, and yields. This study will provide evidence-based recommendations to enhance key soil properties while achieving high wheat production, supporting the development of sustainable agricultural practices in subtropical agroecosystems.

## 2. Results

### 2.1. Effects of N Fertilization on Soil N and C Pools

**Figure 1:**
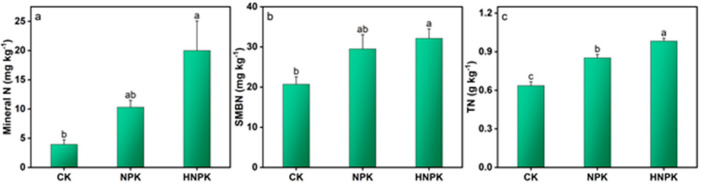
Mineral nitrogen (N, (**a**)), soil microbial biomass N (SMBN, (**b**)), and total N (TN, (**c**)) concentrations in soil under different treatments. Lowercase letters at the top of columns indicate statistical differences among treatments (*p* < 0.05), while error bars denote the standard error (n = 3). Unfertilized control (CK), N fertilization at the rates of 130 kg ha^−1^ (NPK) and 170 kg ha^−1^ (HNPK).

The N fertilization significantly enhanced both soil N and C nutrients as compared to CK (*p* < 0.05). Mineral N content reached its maximum value of 20.01 mg kg^−1^ under HNPK, which was statistically comparable to concentrations observed under NPK fertilization (Figure 1a). TN concentrations were highest under HNPK (0.98 g kg^−1^), representing a significant increase compared to both NPK and CK (Figure 1b). Similarly, SMBN achieved its peak value of 32.15 mg kg^−1^ under HNPK, which was statistically comparable to NPK but significantly exceeded CK (Figure 1c).

**Figure 2:**
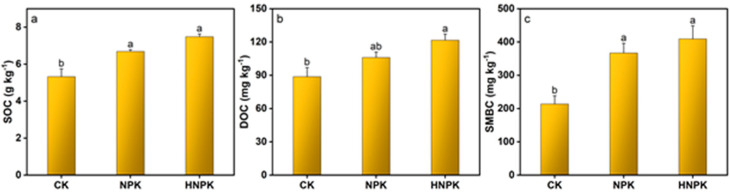
Soil organic carbon (SOC, (**a**)), dissolved organic carbon (DOC, (**b**)), and soil microbial biomass carbon (SMBC, (**c**)) concentrations in soil under different treatments. Lowercase letters at the top of columns indicate statistical differences among treatments (*p* < 0.05), while error bars denote the standard error (n = 3). Unfertilized control (CK), N fertilization at the rates of 130 kg ha^−1^ (NPK) and 170 kg ha^−1^ (HNPK).

Similarly, SOC content was found to be higher under HNPK (7.48 g kg^−1^), which was statistically comparable to NPK but significantly surpassed CK (Figure 2a). The highest DOC concentration (121.60 mg kg^−1^) was found under HNPK, which was comparable to those recorded under NPK but significantly higher than CK (Figure 2b). Furthermore, SMBC reached its highest value of 409.08 mg kg^−1^ under HNPK, demonstrating statistical parity with NPK while exceeding CK (Figure 2c).

### 2.2. Effects of N Fertilization on Plant Nutrients and NUE

**Figure 3:**
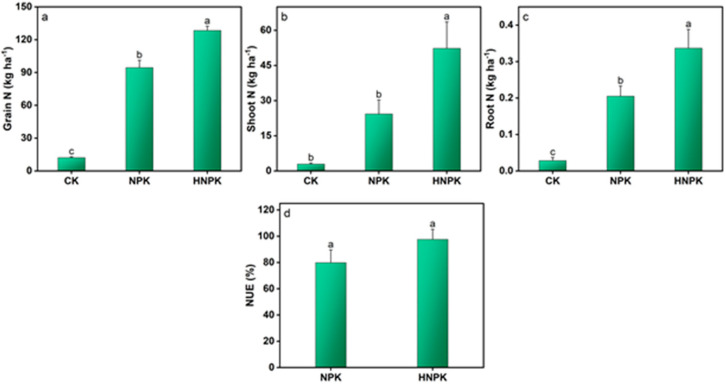
Nitrogen (N) uptake by different components of the wheat plant (**a**–**c**), and N use efficiency (NUE, (**d**)) under different treatments. Lowercase letters at the top of columns indicate statistical differences among treatments (*p* < 0.05), while error bars denote the standard error (n = 3). Unfertilized control (CK), N fertilization at the rates of 130 kg ha^−1^ (NPK) and 170 kg ha^−1^ (HNPK).

The plant N and C nutrients demonstrated substantial increases under N fertilization compared to CK (*p* < 0.05). Grain N uptake was maximized under HNPK (128.46 kg ha^−1^), which was statistically equivalent to NPK (Figure 3a). Conversely, the lowest grain N uptake was recorded under CK. Shoot N uptake achieved its highest value under HNPK (52.31 kg ha^−1^), significantly exceeding both NPK and CK (Figure 3b). Similarly, root N uptake was also maximized under HNPK (0.33 kg ha^−1^), demonstrating significant increases compared to both NPK and CK (Figure 3c). Notably, NUE exhibited no significant differences between NPK and HNPK (Figure 3d).

**Figure 4:**
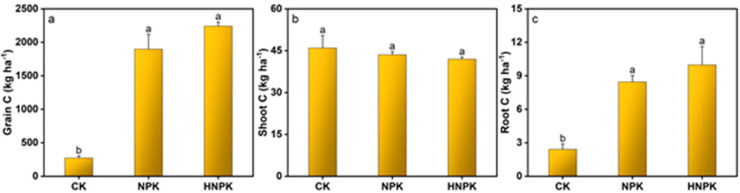
Carbon (C) accumulation by different components of the wheat plant (**a**–**c**) under different treatments. Lowercase letters at the top of columns indicate statistical differences among treatments (*p* < 0.05), while error bars denote the standard error (n = 3). Unfertilized control (CK), N fertilization at the rates of 130 kg ha^−1^ (NPK) and 170 kg ha^−1^ (HNPK).

Moreover, C accumulation by different plant components largely followed similar trends to N uptake. The C accumulation by grain reached its peak under HNPK (2241.85 kg ha^−1^), showing statistical equivalence to NPK while significantly exceeding CK (Figure 4a). In contrast, shoot C accumulation remained statistically comparable across all treatments (Figure 4b). The C accumulation by plant roots was highest under HNPK (9.98 kg ha^−1^), demonstrating statistical parity with NPK while surpassing CK (Figure 4c).

### 2.3. Effects of N Fertilization on Yields and Their Relationship with Soil and Plant Nutrients

**Figure 5:**
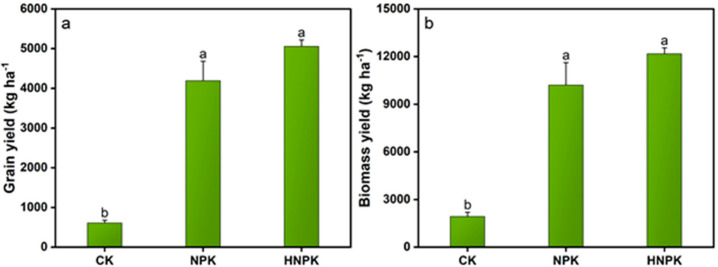
Grain yield (**a**) and biomass yield (**b**) of the wheat crop under different treatments. Lowercase letters at the top of columns indicate statistical differences among treatments (*p* < 0.05), while error bars denote the standard error (n = 3). Unfertilized control (CK), N fertilization at the rates of 130 kg ha^−1^ (NPK) and 170 kg ha^−1^ (HNPK).

Both grain and biomass yields demonstrated significant enhancements under N fertilization compared to CK (*p* < 0.05). Grain yield was maximized under HNPK (5060 kg ha^−1^), showing statistical equivalence to NPK while significantly exceeding CK (Figure 5a). Similarly, biomass yield achieved its highest value under HNPK (12,180.09 kg ha^−1^), again demonstrating statistical parity with NPK while surpassing CK (Figure 5b).

**Figure 6:**
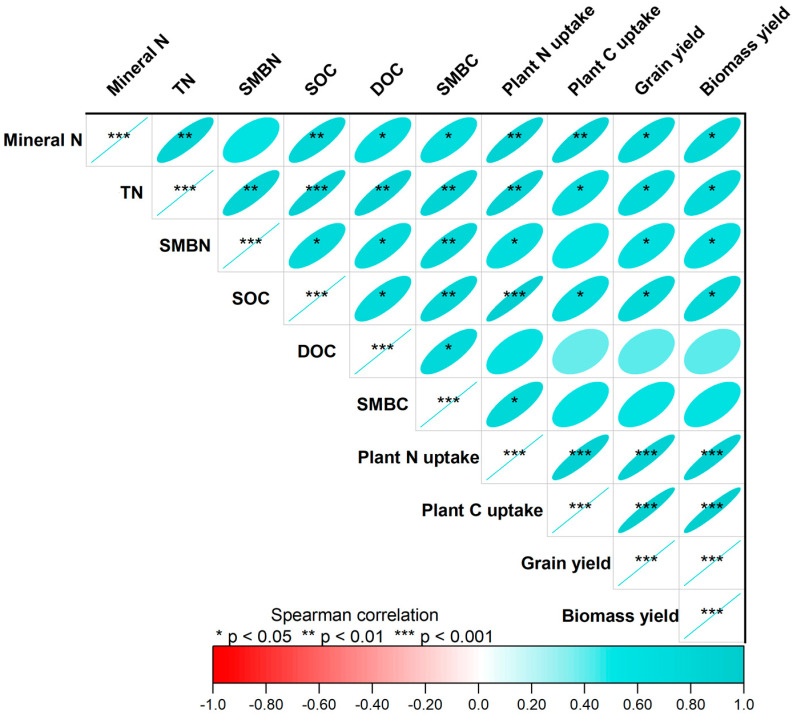
Correlogram showing associations between mineral nitrogen (N), total N (TN), soil microbial biomass N (SMBN), soil organic carbon (SOC), dissolved organic carbon (DOC), soil microbial biomass carbon (SMBC), plant N and C accumulation, and grain and biomass yields of wheat. Positive correlations are displayed in light cyan, with color intensity scaled according to the magnitude of the correlation coefficients.

Spearman correlation analysis revealed that both grain and biomass yields were positively associated with mineral N, TN, SMBN, SOC, and plant N and C uptake parameters (Figure 6). Notably, DOC and SMBC did not exhibit significant relationships with either grain or biomass yields. The mineral N, total N, SMBN, SOC, and SMBC all demonstrated positive correlations with plant total N uptake. In contrast, only mineral N, TN, and SOC showed significant positive associations with total C accumulation by plants.

**Figure 7:**
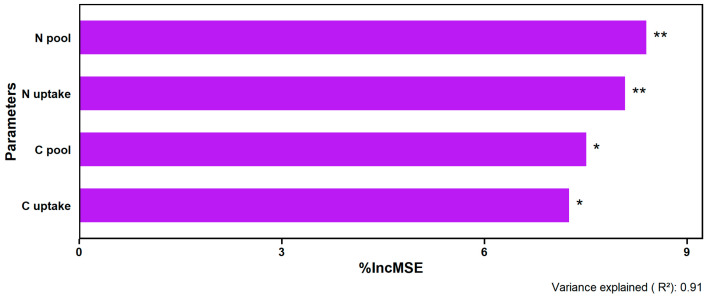
Variable importance from Random Forest model showing the relative influence (%) of soil N and C pools and plant N and C accumulation on wheat yields. Significance levels (** *p* < 0.01; * *p* < 0.05) reflect the permutation-based percentage increase in the mean squared error (%IncMSE).

The Random Forest model explained 91% of the variance in average wheat grain and biomass yields, and identified the soil N pool and plant N uptake as the primary predictors (%IncMSE > 8). Additionally, both the soil C pool and plant C accumulation also emerged as modestly significant predictors of yield performance (Figure 7).

## 3. Discussion

### 3.1. N Fertilization Effects on Soil N and C Pools

The significant increases in soil N and C nutrients under different fertilization rates observed in the present study highlight the complex interactions between nutrient management and soil biogeochemical processes. The enhanced mineral N content under both NPK and HNPK likely reflects the direct input of synthetic N fertilizers, combined with improved N mineralization from increased soil organic matter pools [28,29]. The significantly higher total N concentrations (0.98 g kg^−1^) recorded under HNPK indicate that sustained N inputs over the long-term period have facilitated gradual N accumulation in the soil matrix, potentially through enhanced formation of organo-mineral complexes and increased microbial N immobilization [30,31,32]. Similarly, the significant increases in microbial biomass N under both NPK and HNPK as compared to CK (Figure 1b) may be attributed to the enhanced microbial activity and population growth, which is consistent with previous findings that N availability often limits microbial growth in agricultural soils [33,34]. The increase in microbial biomass N is particularly important because soil microorganisms serve as both a sink and source of nutrients, regulating N availability to plants through mineralization–immobilization turnover processes [35,36,37]. The enhanced microbial biomass likely contributes to improved nutrient cycling efficiency and may partly explain the sustained crop productivity we observed under both fertilized treatments.

The observed increases in soil N pools demonstrate the effectiveness of N inputs, but understanding the broader implications of fertilization may require examining the underlying mechanisms driving these changes. N fertilization effects operate through interconnected processes within soil microsites, including nutrient transformations in the rhizosphere zones, microbial activity within soil aggregates, and gas exchange in pore spaces [38,39]. Together, such processes influence N and C dynamics by modifying plant–soil–microbe interactions and facilitating organo-mineral complex formation, creating feedback loops that regulate nutrient availability and stabilize soil organic matter [40].

These interconnected soil processes are particularly evident in C dynamics, where the positive response of SOC to N fertilization in the present study (Figure 2a), while seemingly counterintuitive given concerns about synthetic fertilizers’ effects on soil properties, aligns with several recent studies demonstrating that adequate N supply can enhance C sequestration in agricultural systems [12,41,42]. Notably, the initial soil organic matter at the study site was very low (Table 1), creating a high potential for C sequestration that likely amplified the soil’s responsiveness to N fertilization and SOC accrual. Multiple interconnected mechanisms could explain the enhanced C storage under N fertilization, including increased crop residue production and root biomass under fertilized conditions that provide greater C inputs to the soil system [43,44], and the stoichiometric relationship between N and C in soil organic matter that enables N availability to enhance the stabilization of C compounds through microbial processing and formation of stable organo-mineral associations [45,46,47]. The increased microbial biomass C we observed under N fertilization provides direct evidence for enhanced C processing mechanisms, as enhanced N availability stimulates microbial growth and activity, leading to more efficient C processing and incorporation into soil organic matter pools [48,49]. Additionally, the elevated DOC concentrations under NPK and HNPK likely reflect increased microbial activity and enhanced decomposition of organic matter, which can mobilize previously stabilized C compounds [50,51]. From a climate change perspective, while higher DOC concentrations in soil could potentially elevate greenhouse gas emissions due to enhanced microbial respiration and decomposition processes that convert organic C to CO_2_ [52,53,54], the enhanced SOC accrual represents a durable C sink that likely reduces net CO_2_ emissions from the soil, thereby contributing to long-term climate mitigation through stable C sequestration in agricultural systems [55,56].

### 3.2. N Fertilization Effects on Plant Nutrient Uptake and NUE

The substantial increases in plant N uptake across all plant components under fertilized treatments as compared to CK (Figure 3) demonstrate the effectiveness of synthetic N fertilizers in enhancing nutrient availability to crops. The particularly pronounced response in grain N uptake under both NPK and HNPK reflects the strong sink strength of developing grains and the plant’s ability to mobilize N from vegetative tissues during grain filling [57,58]. The enhanced N uptake by grain likely contributes to improved grain protein content, which is crucial for nutritional quality in wheat-based food systems. The higher shoot N uptake under HNPK compared to NPK suggests that higher N availability extends the period of active N absorption and may enhance the plant’s capacity to maintain the photosynthetic apparatus longer during grain development [59,60,61]. The extended N uptake period could be beneficial under the subtropical monsoon climate conditions of the study site, where variable precipitation patterns may affect nutrient availability during critical growth stages [62,63]. Similarly, the significant increases in root N uptake under HNPK indicate that higher N availability may stimulate root growth and activity, potentially improving the plant’s capacity for nutrient and water acquisition [64,65].

The C accumulation patterns by different components of the plant largely mirrored N uptake trends, reflecting the fundamental coupling between N and C metabolism in plants [66,67]. The enhanced grain C accumulation under both NPK and HNPK likely results from improved photosynthetic capacity due to adequate N supply for chlorophyll synthesis and enzyme production [68,69]. The relatively stable shoot C accumulation across all treatments suggests that C allocation patterns may shift under different N availability levels, with fertilized plants potentially allocating more C to grain production rather than maintaining vegetative biomass [70,71].

Notably, the results showed that both NPK and HNPK resulted in comparable NUE (Figure 3d). The extra 40 kg N ha^−1^ in HNPK was not proportionally reflected in biomass N, illustrating diminishing returns at high N rates and consistent with the concept of N saturation in agroecosystems [72,73,74]. The observed plateau in NUE could stem from genetic limits on N assimilation in the chosen wheat cultivar, suboptimal timing or method of application at higher rates, or site-specific factors such as soil pH and moisture conditions [75,76]. From an environmental perspective, the comparable NUE under both NPK and HNPK indicates that beyond a certain threshold, excessive N inputs offer limited agronomic benefits while likely elevating the risk of environmental losses through multiple pathways including N_2_O emissions, nitrate leaching, ammonia volatilization, and denitrification processes [26,27]. Such environmental risks are particularly heightened during intense monsoon rains when soil saturation and anaerobic conditions promote N_2_O production and facilitate nutrient transport to water bodies, thereby contributing to both groundwater contamination and climate change through gas emissions [10,77,78].

### 3.3. N Fertilization Effects on Yields and Their Relationship with Plant and Soil Nutrients

The significantly higher yields under both fertilized treatments compared to CK (Figure 5) highlight the central role of synthetic N in sustaining wheat productivity. However, the comparable grain yields between NPK and HNPK, despite the 31% increase in the N application rate, suggest that NPK may represent a more economically and environmentally sustainable approach for this cropping system. Similarly, the enhanced biomass production under both fertilized treatments indicates improved overall plant growth and development, which can have cascading effects on the entire cropping system. The higher biomass production typically translates to increased crop residue availability, which may enhance soil organic matter inputs and could improve soil structure and water retention capacity [79,80,81]. The positive feedback loop between N fertilization, biomass production, and soil property improvement in the present study may partly explain the sustained productivity we observed after the long-term experimental period. Nevertheless, the lack of additional yield gains under HNPK compared to NPK demonstrates that excessive N applications beyond 130 kg ha^−1^ do not further improve crop performance. This suggests that farmers can maintain NPK-level inputs without sacrificing productivity, thereby reducing fertilizer costs while also mitigating environmental pollution risks.

The highly significant positive correlations between soil N and C pools, plant nutrient uptake, and crop yields we observed in the present study (Figure 6) highlight the interconnected nature of soil–plant nutrient cycling processes. Such relationships demonstrate that key soil properties, particularly SOC and microbial biomass, serve as reliable predictors of system productivity and offer potential for developing site-specific nutrient management strategies. Additionally, the positive associations between soil microbial biomass and plant N uptake may emphasize the fundamental role of soil microorganisms in facilitating nutrient mineralization, transformation, and plant availability [82,83]. Enhanced microbial activity under fertilized conditions probably accelerates organic matter decomposition and nutrient mineralization, increasing the availability of nutrients to plants [84,85]. Microbially mediated nutrient cycling may be particularly important in the purple soil of the present study, where the relatively high pH and calcium carbonate content could influence nutrient availability and microbial community dynamics [86,87].

The findings of our study demonstrate that moderate synthetic N fertilization achieves an optimal equilibrium between wheat productivity, enhancement of soil properties, and environmental sustainability in subtropical agroecosystems. The absence of further yields or soil property improvements under higher N inputs, coupled with unchanged NUE, validates our hypothesis that both moderate and high N rates can confer comparable benefits. These results provide evidence-based recommendations for achieving soil maintenance goals while maintaining high wheat productivity in intensive cropping systems, thereby supporting the development of sustainable and climate-smart agricultural practices in subtropical agroecosystems that align with environmental protection goals.

## 4. Materials and Methods

### 4.1. Experimental Site Characteristics

**Figure 8:**
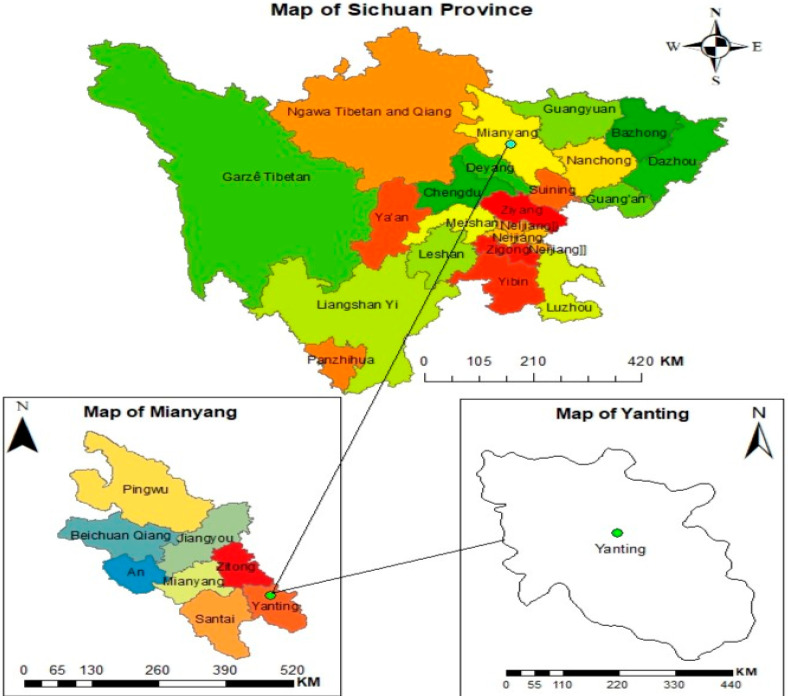
Map of the study area.

This study was conducted at the Yanting Agro-ecological Experimental Station of Purple Soil, operated by the Chinese Academy of Sciences. The station is located at 31°16′ N, 105°28′ E in Linshan village, Mianyang city, Sichuan Province, within the southwestern region of China (Figure 8). The station operates as an integral component of the Chinese Ecosystem Research Network (CERN), where a long-term field experiment has been continuously maintained since plot establishment in 2003. The subtropical monsoon climate is characterized by annual precipitation averaging 826 mm, with the majority concentrated during the wet season spanning June through September. The mean annual air temperature is 17.3 °C, with approximately 290 frost-free days per year.

The experimental soil is locally designated as ‘purple soil’ based on its distinctive coloration and is taxonomically classified as Pup-Orthic Entisol according to the Chinese Soil Taxonomy system, or alternatively as Eutric Regosols under the FAO Soil Classification framework [27]. The soil type represents a prevalent agricultural substrate throughout the upper Yangtze River basin within the Sichuan Basin region. Initial soil properties before conducting this long-term field experiment are presented in Table 1 [9].

**Table 1:** Initial soil properties before the field experiment.

Parameter	Value	Unit
pH	8.30	
Mineral N	42.29	mg kg^−1^
Total N	0.62	g kg^−1^
Unsaturated hydraulic conductivity	16.80	mm h^−1^
Organic matter	8.75	g kg^−1^

### 4.2. Experimental Design

**Figure 9:**
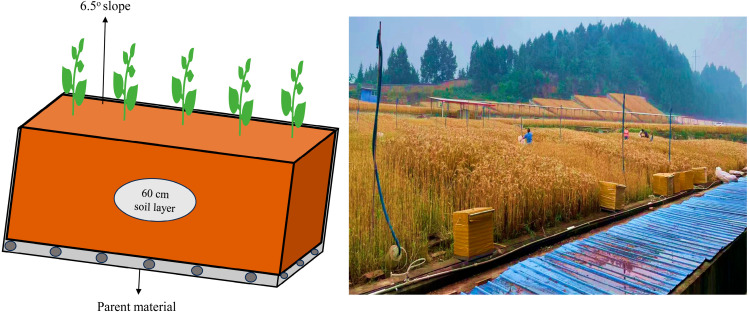
Illustration (**left**) of the long-term experimental plot and photo (**right**) of the wheat harvesting stage.

The experiment was conducted with a completely randomized design incorporating three replications within multi-purpose lysimeter plots (Figure 9). To ensure baseline uniformity across all experimental units, soil was thoroughly homogenized before plot establishment. Individual plots measured 8 × 4 m with a soil profile depth of 60 cm, with concrete barriers installed between plots to eliminate lateral water and nutrient movement. The cropping system employed an annual rotation sequence consisting of rain-fed winter wheat followed by summer maize. Three representative treatments, continuously applied since 2003, were selected for this study: (1) unfertilized control (CK); (2) moderate synthetic N fertilization (NPK: 130 kg N ha^−1^ applied to wheat); and (3) higher synthetic N fertilization (HNPK: 170 kg N ha^−1^ applied to wheat). The application of N at the rate of 130 kg ha^−1^ (NPK) is considered moderate as it is the local recommended dose for wheat in the double cropping system [88]. We increased this dose by 31% (HNPK) to assess the long-term impacts of higher N inputs on different soil properties and crop productivity. Phosphorus and potassium applications were maintained at consistent rates across both fertilized treatments: 90 kg ha^−1^ (P) and 36 kg ha^−1^ (K), respectively. The synthetic nutrient sources comprised urea for N, calcium superphosphate for phosphorus, and potassium chloride for potassium supply. Fertilizer incorporation was achieved through rotary tillage to ensure uniform distribution throughout the plow layer (0–20 cm depth). Winter wheat cultivation started in early November 2023 with a seeding rate of 225 kg ha^−1^, followed by harvest in early May 2024. Weed management was performed manually during early crop development stages. The absence of significant insect and pathogen pressure eliminated the need for pesticide or insecticide applications.

### 4.3. Soil Sampling and Analysis

Soil samples were collected at the harvest stage of wheat on 10 May 2024, upon the physiological maturity of the crop. Bulk soil samples were obtained from 0–20 cm depth using a specialized auger with a 15 cm diameter. Five randomly distributed samples were collected from each plot and combined through manual homogenization to create a representative composite sample of approximately 250 g. Samples were appropriately labeled and immediately transported to the analytical laboratory at the Yanting Agro-ecological Experimental Station of Purple Soil. Each composite sample was subsequently divided into two portions: one fraction was air-dried and passed through a 2 mm sieve for total N (TN) and SOC determination, while the other portion was preserved at 4 °C for analysis of dissolved organic carbon (DOC), mineral N, and soil microbial biomass N (SMBN) and C (SMBC).

TN and SOC quantification employed combustion analysis using an Elemental Analyzer (Vario Macro cube, Elementar, Germany) once air-dried and sieved samples underwent pretreatment with 10 mL of 0.5 M HCl solution for 48 h to eliminate excessive carbonates before analysis [89]. DOC determination followed the water extraction methodology [90]. Briefly, field-moist samples (5 g) were extracted with 30 mL of ultra-pure water (1:6 *w*/*v* ratio). The mixture underwent mechanical agitation for 1 h at 180 rpm, followed by filtration through 0.45 μm membrane filters to remove particulate matter. DOC concentrations were subsequently measured using UV-spectrophotometry (UV-2450, Shimadzu, Japan) [91]. Mineral N analysis utilized spectrophotometric methods after extracting 5 g of fresh soil samples with 25 mL of 2 M KCl solution (1:5 *w*/*v* ratio) [92].

SMBC and SMBN were determined using the chloroform (CHCl_3_) fumigation extraction technique [93]. Briefly, field-moist subsamples (10 g) were prepared in duplicate, with one set extracted directly using 50 mL of 0.5 M K_2_SO_4_ (1:5 *w*/*v* ratio), while the parallel set underwent 24 h fumigation with CHCl_3_ in vacuum chambers within 25 mL glass beakers before extraction with the same solution. SMBC and SMBN values were calculated as the difference in DOC or dissolved N between fumigated and non-fumigated samples, with appropriate correction factors of 0.45 and 0.54, respectively [94,95].

### 4.4. Plant Nutrient Content and Yield Determination

At complete physiological maturity of the wheat crop, plants were manually harvested from a 1 m^2^ area within each plot. Wheat grains were separated from vegetative biomass to enable independent determination of grain and total biomass yields. All plant components (shoots, roots, and grains) were oven-dried at 60 °C for 48–72 h until constant mass was achieved and then analyzed for N and C content using combustion analysis with an Elemental Analyzer (Vario Macro cube, Elementar, Germany).

Additionally, N use efficiency (NUE) was calculated using the following formula [77]:

$$NUE\;(\%)=\frac{\left(N_{1}-N_{0}\right)}{F}\times 100$$

where N_1_ represents total N uptake (kg N ha^−1^) by plants in fertilized treatments, N_0_ denotes total N uptake (kg N ha^−1^) by plants in the unfertilized control, and F indicates the applied N fertilizer rate (kg N ha^−1^).

### 4.5. Statistical Analysis

Treatment effects on soil N and C pools, plant N and C uptake across different plant components, NUE, and crop yields were evaluated using one-way ANOVA followed by Tukey’s HSD post hoc test (*p* < 0.05). These analyses were performed using the ‘agricolae’ package (v1.3-7) in R v4.5.0 [96]. Pairwise Spearman correlation analyses between soil N and C pools, total plant N and C uptake, grain yield, and biomass yield were conducted using the ‘psych’ R package (v2.4.12). The Random Forest model, with 500 permutations and 500 trees, was used to evaluate the relative importance of different predictors on wheat yields via the ‘rfPermute’ R package (v2.5.2).

## 5. Conclusions

This study demonstrates that moderate synthetic N input (130 kg N ha^−1^) is sufficient for maintaining high wheat productivity while enhancing key soil properties in long-term subtropical agroecosystems. Increasing N rate beyond this level did not further improve crop yields or N use efficiency, indicating that excessive inputs provide no additional agronomic benefits while potentially compromising resource use efficiency in high-input systems. The findings provide long-term empirical evidence supporting moderate synthetic N fertilization as a sustainable strategy that balances productivity with environmental security in intensive cropping systems. Future research should prioritize validation of optimal N rates across diverse edaphic and climatic contexts, with concurrent assessment of actual environmental losses and greenhouse gas emissions to comprehensively evaluate the environmental implications of moderate fertilization strategies for sustainable agricultural intensification.

## Data Availability

Data will be provided on demand.
